# Interrelationships of cervical spine sagittal alignment and whole spinopelvic alignment under implications of musculoskeletal health among independent elderly women in Taiwan: A cross-sectional study

**DOI:** 10.1371/journal.pone.0312082

**Published:** 2024-10-31

**Authors:** Tzai-Chiu Yu, Wen-Tien Wu, Ru-Ping Lee, Ing-Ho Chen, Jen-Hung Wang, Shu-Hui Wen, Kuang-Ting Yeh

**Affiliations:** 1 Department of Orthopedics, Hualien Tzu Chi Hospital, Buddhist Tzu Chi Medical Foundation, Hualien, Taiwan; 2 Institute of Medical Sciences, Tzu Chi University, Hualien, Taiwan; 3 School of Medicine, Tzu Chi University, Hualien, Taiwan; 4 Department of Medical Research, Hualien Tzu Chi Hospital, Buddhist Tzu Chi Medical Foundation, Hualien, Taiwan; 5 Department of Public Health, Tzu Chi University, Hualien, Taiwan; 6 Graduate Institute of Clinical Pharmacy, Tzu Chi University, Hualien, Taiwan; National Trauma Research Institute, AUSTRALIA

## Abstract

**Introduction:**

Older women are at increased risk of spinal misalignment and its associated complications. This study investigated the influence of age, grip strength, and various sagittal spinal parameters on spinal alignment. The results indicate the need for comprehensive management strategies.

**Methods:**

This cross-sectional study included 200 older women who underwent bone health evaluations at the orthopedic department of a hospital. The study participants underwent dual-energy X-ray absorptiometry, grip strength measurement, and full-length spine radiography. Clinical and radiographic parameters were analyzed through Pearson correlation and linear regression analyses.

**Results:**

Significant correlations were identified between grip strength and spinal parameters such as C7 slope and global tilt, indicating that muscle strength affects spinal alignment. Advanced age was associated with changes in sagittal spinal parameters, indicating that changes occur in body compensation over time. Furthermore, pelvic parameters such as pelvic tilt and sacral slope were significantly correlated with spinal curvature, indicating their critical roles in maintaining spinal stability.

**Conclusions:**

This study revealed the critical roles of muscle strength and pelvic alignment in the management of spinal health in older women with low bone mass. Targeted interventions for increasing muscle strength, correcting posture, and achieving hormonal balance can notably improve spinal stability and reduce the risk of associated complications. Longitudinal studies should be conducted to validate and refine the intervention strategies and to extend the study findings.

## Introduction

Due to their association with increased risks of fracture and related complications, osteoporosis and osteopenia are major public health concerns globally, particularly among older women. Globally, osteoporosis affects approximately 10% of women aged ≥ 60 years and 40% of women aged ≥ 80 years [1]. A recent study reported that in Taiwan specifically, the prevalence of osteoporosis in women aged >50 years is approximately 25%, and an additional 45% of these women may have osteopenia [2]. These conditions are characterized by significant decreases in bone mineral density (BMD) after menopause, which contribute to an increased risk of fractures [3]. However, the risk of fractures is not solely determined by bone density; changes in spinal alignment also play a crucial role in the risk of fractures, and these changes may influence balance, gait, and overall fall risk [4]. Spinal alignment in older adults should be assessed to determine the biomechanical changes accompanying aging and bone mass reduction [5]. Global sagittal balance parameters, such as sagittal vertical axis (SVA) and global tilt (GT), are strongly correlated with clinical outcomes in older patients [6]. These parameters reflect overall posture and are crucial predictors of mechanical efficiency and mobility limitations in older patients. A multicenter study reported that SVA increases by an average of 3 mm per year in individuals aged > 50 years, with more rapid progression in those with osteoporosis [7]. Cervical lordosis (CL) plays a critical role in balancing the head and maintaining its alignment with respect to the rest of the spine. Alterations in cervical angles can lead to significant biomechanical stress and major compensatory changes in other spinal regions [8]. Furthermore, thoracic kyphosis (TK) and lumbar lordosis (LL) determine the overall curvature and posture of the spine [9]. Moreover, pelvic parameters such as pelvic incidence (PI), sacral slope (SS), and pelvic tilt (PT) are integral to maintaining sagittal balance and are correlated with spinal health and mobility [10]. Recent international studies have highlighted the complex interplay between these spinal and pelvic parameters in different populations. A large-scale study found that decreased LL was associated with a 1.5-fold increase in the risk of osteoporotic vertebral fractures in women aged > 65 years [11]. Another study similarly demonstrated that for every 10° increase in TK, the risk of injurious falls increases by 22% among older adults [12]. Despite these findings at the global scale, comprehensive data on spinal alignment patterns specific to Taiwanese older adults, particularly in relation to bone density, are lacking. These relationships should be determined for older adults in Taiwan for developing targeted interventions and preventive strategies to reduce the risk of fall-related injuries and to improve quality of life for older adults with low bone mass.

To address the aforementioned knowledge gap, the current study explored the correlations between clinical and radiographic parameters, including BMD, grip strength, and various spinal alignment measures, in older women with low bone mass. Analysis of the associations between these parameters can provide comprehensive insights into the influence of spinal alignment on bone health and may be useful for clinicians aiming to increase functional independence in this demographic.

We hypothesize that changes occur in spinal alignment with age, with potential implications for developing clinical management strategies and for improving quality of life. Our research objectives are to characterize spinal alignment parameters across different age groups; investigate correlations between spinal alignment, bone density, and muscle strength; and identify age-related changes in spinal alignment and their potential clinical implications.

## Methods

This study was conducted with the approval of the Institutional Review Board. All ethical considerations were appropriately addressed (IRB 107-255-B; date of approval, August 1, 2019). Written informed consent was obtained from all participants, and the process was thoroughly documented and witnessed to ensure compliance with ethical standards.

### Patient selection

Women aged 60 years or older undergoing bone health evaluations between August 1, 2019, and July 31, 2020, at the orthopedic department of our hospital were recruited. Women were recruited if they had no obvious neck or back pain, had a normal walking status, and had a Barthel index functional score of > 80. Women were excluded if they had a history of spinal or hip surgery, spinal tumors, or spinal infections, inflammatory arthritis, congenital spinal deformities, or neuromuscular disorders affecting the spine. Additionally, women were excluded if they were unable to stand independently for the required imaging procedures or had any cognitive impairment that interfered with their ability to follow instructions, indicating significant dependency in activities of daily living. In addition, to minimize the effects of potential confounding factors on spinal alignment, we excluded participants with a leg length discrepancy of > 2 cm. These exclusion criteria were implemented to ensure that the study population represented independent older women without major spinal pathologies, lower limb discrepancies, or other conditions that could significantly alter natural spinal alignment. By applying these criteria, we aimed to enhance the validity and reliability of our findings regarding the relationships between spinal alignment and musculoskeletal health in this specific population. All the recruited women participated voluntarily and provided informed consent.

### Radiographic measurement

Upon enrollment into this study, participants underwent dual-energy X-ray absorptiometry for BMD measurement. Full-length standing anteroposterior and lateral radiographs were obtained for the measurement of various spinal alignment parameters. These parameters included global sagittal spinal parameters (GT and SVA) and regional parameters [upper cervical lordosis (UCL), middle cervical lordosis (MCL), lower cervical lordosis (LCL), C7 slope, upper thoracic kyphosis (UTK), lower thoracic kyphosis (LTK), upper lumbar lordosis (ULL), lower lumbar lordosis (LLL), PI, SS, and PT].

In this study, the sagittal spinal parameters were precisely defined and measured using standardized techniques. GT is the angle between a line extending from C7 to the center of the sacrum and another line connecting the center of the sacrum to the center of the femoral heads. SVA is the horizontal distance from the posterior superior sacral end plate to a plumb line dropped vertically from the centroid of the C7 vertebral body. For detailed measurement, CL is segmented into three areas: UCL, MCL, and LCL. UCL is the angle formed between the cranial line (C0) and the lower end plate of C2. MCL is the angle formed between the lower end plate of C2 and the lower end plate of C5. LCL is the angle formed between the lower end plate of C5 and the lower end plate of C7. The C7 slope is measured as the angle between a horizontal reference line and a line parallel to the upper end plate of C7. TK is divided into UTK and LTK, which are measured as the angles between the end plates of T5 and T9 and between the end plates of T9 and T12, respectively. For LL, ULL is the angle formed between the lower end plates of L1 and L4, and LLL is the angle between the lower end plate of L4 and the upper end plate of S1. SS is the angle between the upper end plate of S1 and a horizontal line. PT is the angle formed between a vertical line and a line from the midpoint of the sacral end plate to the femoral rotational axis; it captures the pelvic orientation relative to the lower extremities. Obtaining precise measurements is critical for evaluating spinal alignment and determining its biomechanical implications. Three physicians independently obtained the measurements, with intraclass correlation coefficients of 0.86 and 0.80 for intraobserver and interobserver reliability, respectively.

### Statistical analysis

Statistical analyses were performed using IBM SPSS Statistics for Windows (version 23.0; IBM, Armonk, NY, USA). The Kolmogorov–Smirnov test was used to determine whether the data followed a normal distribution. An independent *t* test or Wilcoxon rank-sum test was used to identify the significant differences in the means of clinical and radiographic parameters between two age groups (i.e., < 70 vs. ≥ 70 years) depending on the normality of the data. The chi-squared test was used to evaluate the association between categorical variables. Pearson correlation and linear regression (simple and multiple) analyses were employed to assess the relationships between clinical and radiographic parameters. Participants with missing data were excluded from the analysis. All tests were two-sided, with significance set at *p* < 0.05.

### Sample size estimation

We used G*Power 3.1.9.2 to calculate the required sample size for this study. In the multiple linear regression analysis of the association between cervical spine sagittal alignment and clinical or radiographic parameters, we set the effect size as 0.12, α as 0.05, power (1 − β) as 0.80, and number of predictors as 22. With these values, the minimum sample size was estimated to be 199.

## Results

Table 1 presents the demographics of the 200 older women with low bone mass who were recruited into this study. The participants were categorized into two age groups: those aged < 70 years and those aged ≥ 70 years. The younger group (age < 70 years) comprised 115 participants, with an average age of 64.10 (± 4.26) years, and the older group (age ≥ 70 years) comprised 85 participants, with an average age of 76.32 (± 4.80) years. For the entire cohort, the overall mean age was 69.29 (± 7.54) years (Table 1). The menopausal period was significantly longer in the older group than in the younger group, with average durations of 26.26 (± 6.84) years and 14.61 (± 6.08) years, respectively. Slight differences were noted in body mass index (BMI) and grip strength between the groups. The younger group had a slightly higher average grip strength (20.52 ± 4.24) than did the older group (19.02 ± 4.60). Significant differences are also observed in average T-scores between the younger group (−1.55 ± 0.71) and the older group (−1.92 ± 0.86; Table 1). Mean values and standard deviations were calculated for several spinal alignment parameters, including CL, TK, and LL, and related measures, such as SS, PI, and PT. These parameters reflect variations in spinal health across different age groups. Notably, significant age-related changes were found in SVA, GT, and LTK, indicating that aging affects spinal alignment and posture (Table 1).

**Table 1:** Demographics of older women with low bone mass (n = 200).

Item	Age Group			*p* value
	< 70 y/o	≧ 70 y/o	Total	
N	115	85	200	
Age‡	64.10 ± 4.26	76.32 ± 4.80	69.29 ± 7.54	< 0.001*
Menopause period‡	14.61 ± 6.08	26.26 ± 6.84	19.56 ± 8.61	< 0.001*
BMI‡	24.15 ± 3.45	24.25 ± 3.80	24.19 ± 3.59	0.843
Grip strength‡	20.52 ± 4.24	19.02 ± 4.60	19.88 ± 4.45	0.018*
Average T score†	-1.55 ± 0.71	-1.92 ± 0.86	-1.70 ± 0.80	0.001*
Barthel index‡	87.06 ± 3.67	85.42 ± 4.06	86.37 ± 3.92	0.003*
Coronal malalignment (%)※	29 (25.2%)	22 (25.9%)	51 (25.5%)	0.915
GT‡	18.62 ± 7.47	21.83 ± 10.38	19.99 ± 8.94	0.012*
SVA‡	32.05 ± 23.22	40.98 ± 27.97	35.85 ± 25.66	0.015*
PT‡	16.76 ± 5.83	19.19 ± 8.54	17.79 ± 7.19	0.018*
UCL‡	36.50 ± 8.73	35.78 ± 8.08	36.20 ± 8.45	0.553
MCL‡	2.84 ± 7.01	5.66 ± 7.52	4.04 ± 7.34	0.007*
LCL‡	6.24 ± 7.39	5.64 ± 7.31	5.98 ± 7.34	0.566
CL‡	45.59 ± 10.20	47.08 ± 11.50	46.22 ± 10.77	0.334
UTK‡	-17.51 ± 8.33	-18.20 ± 7.18	-17.80 ± 7.85	0.543
LTK‡	-4.19 ± 6.15	-8.12 ± 6.99	-5.86 ± 6.79	< 0.001*
TK†	-21.70 ± 10.25	-26.32 ± 8.92	-23.66 ± 9.95	0.001*
ULL‡	15.18 ± 13.37	17.02 ± 12.02	15.96 ± 12.82	0.316
LLL†	29.26 ± 7.98	28.30 ± 10.77	28.85 ± 9.26	0.470
LL‡	44.43 ± 13.82	45.32 ± 12.44	44.81 ± 13.22	0.641
SS‡	34.14 ± 9.83	32.92 ± 9.04	33.62 ± 9.50	0.368
PI ‡	50.91 ± 12.18	52.10 ± 12.10	51.41 ± 12.13	0.492

Data are presented as n or mean ± standard deviation.

*p-value < 0.05 was considered statistically significant after test.

†: independent *t* test

‡: Wilcoxon rank-sum test; ※: chi-squared test.

Abbreviations: UCL, upper cervical lordosis; MCL, middle cervical lordosis; LCL, lower cervical lordosis; CL, cervical lordosis; MP, menopausal period; BMI, body mass index; GT, Global tilt; SVA, sagittal vertical axis; UTK, upper thoracic kyphosis; LTK, lower thoracic kyphosis; ULL, upper lumbar lordosis; LLL, lower lumbar lordosis; SS, sacral slope; PI, pelvic incidence; PT, pelvic tilt.

Table 2 presents the results of the Pearson correlation analysis of various parameters. This study investigated the correlations between the menopausal period, BMI, grip strength, average T-score, and various spinal alignment parameters, including CL (UCL, MCL, and LCL), TK (UTK and LTK), LL (ULL and LLL), and related measures (C7 slope, PT, and SVA). The Pearson correlation analysis revealed a significant negative correlation between menopausal period and grip strength (r = −0.188, *p* = 0.008); this finding indicates that a lower muscle strength is observed in patients with a longer menopausal period. Additionally, menopausal period was negatively correlated with LTK (r = −0.236, *p* = 0.001) and positively correlated with MCL (r = 0.215, *p* = 0.002); this finding suggests that menopausal period differently influences the segment of CL (Table 2). BMI was significantly positively correlated with C7 slope (r = 0.221, *p* = 0.002) and significantly negatively correlated with LTK (r = −0.263, *p* < 0.001); this finding indicates that body mass affects the spinal curvature and alignment. Grip strength was notably correlated with several spinal parameters. Specifically, grip strength exhibited a strong negative correlation with C7 slope (r = −0.325, *p* < 0.001) and a positive correlation with ULL (r = 0.195, *p* = 0.006); these results indicate that higher muscle strength is associated with greater lumbar or cervical spinal alignment. Furthermore, the average T-score, which reflects bone density, was negatively correlated with LCL (r = −0.190, *p* = 0.007) and positively correlated with UTK (r = 0.256, *p* < 0.001); this finding suggests that low bone density might contribute to adverse changes in specific spinal regions, potentially affecting the overall posture and stability (Table 2).

**Table 2:** Pearson correlation analysis (n = 200).

Title 1		Menopausal period	Body Mass Index	Grip Strength	Average T score	Barthel index score	Coronal malalignment	UCL	MCL	LCL	C7 slope	UTK	LTK	ULL	LLL
Menopausal period	Pearson Correlation	1	0.118	-0.188**	-0.124	-0.062	0.068	0.128	0.215**	-0.034	0.130	-0.059	-0.236**	-0.002	-0.180*
	Significance		0.097	0.008	0.081	0.382	0.337	0.070	0.002	0.630	0.067	0.405	0.001	0.977	0.011
Body Mass Index	Pearson Correlation	0.118	1	0.008	0.167*	0.141*	0.099	0.057	0.160*	0.102	0.221**	0.028	-0.263**	-0.052	-0.171*
	Significance	0.097		0.912	0.018	0.046	0.162	0.420	0.024	0.149	0.002	0.692	0.000	0.461	0.016
Grip Strength	Pearson Correlation	-0.188**	0.008	1	0.108	0.230**	0.046	0.039	-0.202**	-0.119	-0.325**	0.195**	0.165*	-0.110	0.061
	Significance	0.008	0.912		0.129	0.001	0.517	0.588	0.004	0.094	0.000	0.006	0.020	0.120	0.393
Average T score	Pearson Correlation	-0.124	0.167*	0.108	1	0.256**	-0.126	0.073	0.064	-0.190**	-0.081	0.256**	0.062	0.008	-0.051
	Significance	0.081	0.018	0.129		0.000	0.076	0.302	0.367	0.007	0.254	0.000	0.386	0.909	0.470
Barthel index score	Pearson Correlation	-0.062	0.141*	0.230**	0.256**	1	-0.052	0.079	-0.063	0.042	0.123	-0.055	-0.049	-0.036	0.074
	Significance	0.382	0.046	0.001	0.000		0.467	0.267	0.377	0.553	0.082	0.437	0.488	0.609	0.301
Coronal malalignment	Pearson Correlation	0.068	0.099	0.046	-0.126	-0.052	1	-0.073	0.050	0.138	0.183**	0.033	-0.170*	-0.128	0.016
	Significance	0.337	0.162	0.517	0.076	0.467		0.306	0.483	0.052	0.009	0.644	0.016	0.071	0.817
UCL	Pearson Correlation	0.128	0.057	0.039	0.073	0.079	-0.073	1	-0.250**	-0.264**	0.049	0.000	-0.026	0.133	-0.065
	Significance	0.070	0.420	0.588	0.302	0.267	0.306		0.000	0.000	0.495	0.997	0.713	0.061	0.364
MCL	Pearson Correlation	0.215**	0.160*	-0.202**	0.064	-0.063	0.050	-0.250**	1	0.004	0.343**	0.003	-0.009	-0.021	-0.001
	Significance	0.002	0.024	0.004	0.367	0.377	0.483	0.000		0.951	0.000	0.967	0.897	0.768	0.994
LCL	Pearson Correlation	-0.034	0.102	-0.119	-0.190**	0.042	0.138	-0.264**	0.004	1	0.498**	-0.381**	-0.075	0.146*	-0.048
	Significance	0.630	0.149	0.094	0.007	0.553	0.052	0.000	0.951		0.000	0.000	0.293	0.039	0.500
C7 slope	Pearson Correlation	0.130	0.221**	-0.325**	-0.081	0.123	0.183**	0.049	0.343**	0.498**	1	-0.465**	-0.217**	0.049	-0.038
	Significance	0.067	0.002	0.000	0.254	0.082	0.009	0.495	0.000	0.000		0.000	0.002	0.495	0.590
UTK	Pearson Correlation	-0.059	0.028	0.195**	0.256**	-0.055	0.033	0.000	0.003	-0.381**	-0.465**	1	-0.083	-0.359**	0.078
	Significance	0.405	0.692	0.006	0.000	0.437	0.644	0.997	0.967	0.000	0.000		0.245	0.000	0.275
LTK	Pearson Correlation	-0.236**	-0.263**	0.165*	0.062	-0.049	-0.170*	-0.026	-0.009	-0.075	-0.217**	-0.083	1	-0.259**	-0.191**
	Significance	0.001	0.000	0.020	0.386	0.488	0.016	0.713	0.897	0.293	0.002	0.245		0.000	0.007
ULL	Pearson Correlation	-0.002	-0.052	-0.110	0.008	-0.036	-0.128	0.133	-0.021	0.146*	0.049	-0.359**	-0.259**	1	-0.316**
	Significance	0.977	0.461	0.120	0.909	0.609	0.071	0.061	0.768	0.039	0.495	0.000	0.000		0.000
LLL	Pearson Correlation	-0.180*	-0.171*	0.061	-0.051	0.074	0.016	-0.065	-0.001	-0.048	-0.038	0.078	-0.191**	-0.316**	1
	Significance	0.011	0.016	0.393	0.470	0.301	0.817	0.364	0.994	0.500	0.590	0.275	0.007	0.000	

* *p* value < 0.05 (double tailed).

** *p* value < 0.01 (double tailed).

Abbreviations: UCL, upper cervical lordosis; MCL, middle cervical lordosis; LCL, lower cervical lordosis; CL, cervical lordosis; MP, menopausal period; BMI, body mass index; GT, Global tilt; SVA, sagittal vertical axis; UTK, upper thoracic kyphosis; LTK, lower thoracic kyphosis; ULL, upper lumbar lordosis; LLL, lower lumbar lordosis; SS, sacral slope; PI, pelvic incidence; PT, pelvic tilt

Table 3 presents the results of the regression analysis of the association of various clinical and radiographic parameters with CL. Crude and adjusted models were employed for this analysis, and the strength of the association of each parameter with CL was determined on the basis of regression coefficients (β), 95% confidence intervals, and *p* values. In the regression analysis, menopausal period exhibited a positive association with CL in both the crude model (β = 0.28, *p* = 0.001) and adjusted models (β = 0.34, *p* < 0.001). This result indicates that a longer menopausal period is correlated with higher CL (Table 3). BMI also exerted a substantial positive effect on CL, with β values of 0.67 (*p* = 0.001) in the crude model and 0.53 (*p* = 0.003) in the adjusted model. This finding indicates that higher BMI is associated with greater CL. By contrast, grip strength initially exhibited a negative association with CL in the crude model (β = −0.46, *p* = 0.007). However, this association became nonsignificant in the adjusted model (β = 0.06, *p* = 0.665). These findings of the adjusted model reflect the complex interplay of muscle strength with spinal alignment (Table 3). The significant associations between both GT and UTK with CL in the adjusted models underscore their crucial roles in the compensatory mechanisms that maintain cervical alignment. Furthermore, both SVA and LLL were significantly correlated with CL in the adjusted model. SVA exhibited a negative association (β = −0.15, *p* = 0.007), whereas LLL exhibited a positive association (β = 0.46, *p* = 0.003). These findings indicate their influence on cervical spinal posture. PI was negatively correlated with CL in the adjusted model (β = −1.21, *p* = 0.002). This result suggests that the pelvis–spine relationship plays a crucial role in maintaining functionally acceptable sagittal alignment (Table 3).

**Table 3:** Clinical or radiographic factors associated with CL (n = 200).

Item	Crude		Adjusted	
	β (95% CI)	*p* value	β (95% CI)	*p* value
Menopause period	0.28 (0.11, 0.45)	0.001*	0.34 (0.20, 0.48)	< 0.001*
BMI	0.67 (0.26, 1.08)	0.001*	0.53 (0.18, 0.88)	0.003*
Grip strength	-0.46 (-0.79, -0.12)	0.007*	0.06 (-0.22, 0.34)	0.665
Average T score	-0.38 (-2.27, 1.50)	0.690	-0.44 (-1.97, 1.09)	0.569
Barthel index	0.13 (-0.25, 0.52)	0.501	-0.003 (-0.31, 0.31)	0.997
Coronal malalignment (Yes vs. No)	1.74 (-1.70, 5.19)	0.319	-1.10 (-3.84, 1.63)	0.428
GT	0.18 (0.01, 0.35)	0.034*	1.26 (0.52, 2.01)	0.001*
SVA	0.04 (-0.02, 0.10)	0.162	-0.15 (-0.26, -0.04)	0.007*
C7 slope	0.84 (0.69, 0.99)	< 0.001*	0.96 (0.77, 1.15)	< 0.001*
UTK	-0.35 (-0.54, -0.17)	< 0.001*	0.41 (0.21, 0.61)	< 0.001*
LTK	-0.12 (-0.34, 0.10)	0.274	0.74 (0.43, 1.05)	< 0.001*
ULL	0.16 (0.04, 0.27)	0.007*	0.56 (0.29, 0.83)	< 0.001*
LLL	-0.10 (-0.26, 0.07)	0.239	0.46 (0.16, 0.77)	0.003*
SS	0.04 (-0.11, 0.20)	0.583	0.88 (0.18, 1.57)	0.013*
PI	0.08 (-0.05, 0.20)	0.219	-1.21 (-1.98, -0.45)	0.002*

Adjusted R^2^ square: 0.53

Data are presented as β (95% CI).

*p-value < 0.05 was considered statistically significant after test.

Abbreviations: UCL, upper cervical lordosis; MCL, middle cervical lordosis; LCL, lower cervical lordosis; CL, cervical lordosis; MP, menopausal period; BMI, body mass index; GT, Global tilt; SVA, sagittal vertical axis; UTK, upper thoracic kyphosis; LTK, lower thoracic kyphosis; ULL, upper lumbar lordosis; LLL, lower lumbar lordosis; SS, sacral slope; PI, pelvic incidence; PT, pelvic tilt.

Table 4 presents the results of crude and adjusted regression analyses for the associations between various clinical and radiographic factors and C7 slope. Notably, the menopausal period initially exhibited a slight positive correlation with C7 slope in the crude model (β = 0.12, *p* = 0.067). However, in the adjusted model, the menopausal period had a significant negative influence on C7 slope (β = −0.13, *p* = 0.005). These results suggest the complex interactions of the menopausal period with other factors. BMI exhibited a positive effect on C7 slope in the crude model (β = 0.48, *p* = 0.002), but its influence became nonsignificant in the adjusted model (β = −0.04, *p* = 0.708). This result indicates that the influence of BMI may be moderated by additional variables (Table 4). Grip strength had a strong negative association with C7 slope in both models, with more pronounced effects noted in the crude model (β = −0.57, *p* < 0.001) than in the adjusted model (β = −0.27, *p* = 0.001). These results indicate that grip strength has a significant influence on spinal curvature. Other notable findings include the strong correlations of SVA, UCL, MCL, and LCL with C7 slope in the adjusted model, highlighting the interconnectedness of cervical and spinal alignment (Table 4). Additionally, UTK and LTK were negatively correlated with C7 slope in both the models, emphasizing the compensatory role of spinal curvature in maintaining the overall posture. SS also exhibited a negative effect in the adjusted model. This result suggests that pelvic alignment plays a crucial role in the overall spinal profile (Table 4).

**Table 4:** Clinical or radiographic parameters associated with C7 slope (n = 200).

Item	Crude		Adjusted	
	β (95% CI)	*p* value	β (95% CI)	*p* value
Menopause period	0.12 (-0.01, 0.25)	0.067	-0.13 (-0.22, -0.04)	0.005*
BMI	0.48 (0.18, 0.78)	0.002*	-0.04 (-0.26, 0.18)	0.708
Grip strength	-0.57 (-0.81, -0.34)	< 0.001*	-0.27 (-0.44, -0.11)	0.001*
Average T score	-0.80 (-2.17, 0.58)	0.254	0.75 (-0.18, 1.68)	0.114
Barthel index	0.25 (-0.03, 0.53)	0.082	0.20 (0.01, 0.38)	0.038*
Coronal malalignment (Yes vs. No)	3.29 (0.82, 5.77)	0.009*	1.02 (-0.64, 2.68)	0.225
GT	0.09 (-0.03, 0.21)	0.148	-0.3 (-0.77, 0.17)	0.207
SVA	0.06 (0.01, 0.10)	0.009*	0.08 (0.02, 0.15)	0.015*
UCL	0.05 (-0.09, 0.18)	0.495	0.28 (0.19, 0.37)	< 0.001*
MCL	0.37 (0.23, 0.51)	< 0.001*	0.43 (0.33, 0.54)	< 0.001*
LCL	0.53 (0.40, 0.66)	< 0.001*	0.44 (0.33, 0.54)	< 0.001*
UTK	-0.47 (-0.59, -0.34)	< 0.001*	-0.42 (-0.53, -0.31)	< 0.001*
LTK	-0.25 (-0.41, -0.09)	0.002	-0.29 (-0.49, -0.09)	0.005*
ULL	0.03 (-0.06, 0.12)	0.495	-0.11 (-0.28, 0.06)	0.221
LLL	-0.03 (-0.15, 0.09)	0.590	-0.01 (-0.21, 0.18)	0.894
SS	-0.13 (-0.24, -0.01)	0.030*	-0.42 (-0.84, 0.01)	0.055
PI	-0.07 (-0.16, 0.02)	0.121	0.29 (-0.19, 0.77)	0.233

Adjusted R^2^ square: 0.68.

Data are presented as β (95% CI).

*p-value < 0.05 was considered statistically significant after test.

Abbreviations: UCL, upper cervical lordosis; MCL, middle cervical lordosis; LCL, lower cervical lordosis; CL, cervical lordosis; MP, menopausal period; BMI, body mass index; GT, Global tilt; SVA, sagittal vertical axis; UTK, upper thoracic kyphosis; LTK, lower thoracic kyphosis; ULL, upper lumbar lordosis; LLL, lower lumbar lordosis; SS, sacral slope; PI, pelvic incidence; PT, pelvic tilt.

## Discussion

Our analysis of demographics and clinical parameters among older women with low bone mass revealed significant age-related differences in grip strength, T-scores, and spinal alignment (Table 1). This study also observed negative correlations between age, grip strength, and T-scores, consistent with findings in other studies that advanced age is associated with reduced musculoskeletal function and decreased bone density [13, 14]. The results indicate the complexity of interactions between spinal alignment and clinical parameters (Table 2). The analysis revealed notable correlations between the menopausal period, grip strength, and spinal parameters, such as C7 slope and GT. These findings underscore the multifaceted influence of aging on spinal health [4, 15]. Interestingly, we observed statistically significant differences in global alignment parameters (SVA and GT) between the groups, although no significant differences were found in lumbar sagittal parameters. This discrepancy highlights the complex influence of spinal and pelvic parameters on spinal balance in older women; the spine functions as an integrated unit. Relative to local lumbar parameters, global parameters such as SVA and GT, which are comprehensive measures of overall sagittal balance, may be more sensitive to subtle, cumulative changes in all segments of the spine and pelvis [16]. Furthermore, our study specifically revealed a notable change in MCL for both groups—the other cervical parameters remained relatively stable. This selective alteration in MCL may be attributed to its unique biomechanical role as a transitional zone between the more-mobile upper cervical spine and the relatively stable lower cervical spine. The susceptibility of MCL to change may indicate that it serves as a compensatory mechanism for maintaining the horizontal gaze and the overall sagittal balance in response to age-related changes at other locations in the spine [17]. Collectively, these findings emphasize that when assessing spinal balance in older adults, both global and local alignment parameters, including regional cervical changes, should be considered. These parameters provide complementary information on the overall posture and spinal health, which can be used for developing comprehensive management strategies for older adults [18–21]. Longitudinal studies should be conducted to elucidate the mechanisms underlying these selective changes and their clinical implications.

The results presented in Tables 3 and 4 provide insights into the clinical and radiographic parameters influencing CL and C7 slope in our cohort of older women. This study demonstrated a significant positive association between the menopausal period and CL adjustment, as indicated by the adjusted β values. This finding suggests complex interactions between menopause and CL adjustment, highlighting the influence of hormonal changes on spinal curvature over time. It aligns with previous research showing similar associations in postmenopausal women and underscores the role of estrogen deficiency in altering spinal biomechanics [21]. Furthermore, this study revealed a negative association between grip strength and C7 slope in both the crude and adjusted models. This finding indicates the correlation between muscle strength and spinal alignment, which is supported by previous findings indicating that decreased muscle strength can lead to detrimental postural changes and increased spinal curvature. Additionally, alterations in spinal alignment parameters, such as GT and SVA, in response to changing biomechanical loads are indicative of the body’s compensatory mechanisms for maintaining balance and function; these mechanisms are crucial for preventing falls [5, 22]. Our findings of changes in the lumbar and thoracic regions, particularly in terms of the relationships of ULL, LLL, UTK, and LTK with C7 slope, are consistent with those in the literature. These results suggest that cervical alignment in these regions is altered to maintain overall spinal integrity [23]. The finding of the current study that pelvic parameters, such as PI and PT, influence spinal curvature is consistent with previous research showing that PT adjustments are crucial for managing lumbar spine load and maintaining sagittal balance [24]. The significant adjustments observed in these parameters, along with the influence of other spine-related factors identified in this study, underscore their role in the dynamics of spinal alignment. These findings suggest that targeted interventions aimed at improving muscle strength, correcting posture, and enhancing pelvic stability could improve spinal health and reduce the risk of fall-related injuries in older adults [25]. Longitudinal studies should be conducted to examine the causal relationships between spinal and pelvic parameters and the effectiveness of targeted interventions for maintaining or improving spinal health in aging populations.

This study provides notable findings regarding the interactions of spinal and pelvic parameters with clinical outcomes. Thus, our findings have key implications for clinical practice and future research. The findings of a positive correlation of the menopausal period with CL and the multifaceted influence of grip strength on spinal alignment indicate that a multidisciplinary approach should be applied for managing spinal health in older women with low bone mass. Studies have indicated that hormonal imbalance is linked to degenerative changes in spinal alignment; thus, hormone replacement therapy may mitigate some of the adverse effects of estrogen deficiency on the structure of the spine [26, 27]. Moreover, the current study discovered strong associations between muscle strength, spinal curvature, and overall posture. Therefore, comprehensive physical therapy including strength training, flexibility exercises, and posture correction strategies should be implemented for improving spinal health. Such therapy can significantly improve balance, reduce the risk of falls, and enhance the quality of life in this vulnerable population [28]. Additionally, integrating nutritional counseling into these interventions can improve bone health, muscle mass, and overall wellbeing, thus enhancing their effectiveness [29]. Future studies should determine whether such interventions can alter the course of spinal degeneration and improve clinical outcomes in older populations. Moreover, studies should investigate the genetic and environmental factors that contribute to variations in spinal and pelvic alignment among older women; such investigations would enable the development of personalized approaches to geriatric care [30]. Targeted therapies based on individual risk profiles can be developed for addressing specific needs. Such precision health-care strategies can help in maintaining mobility and independence in older adults. With an improved understanding of the complex interactions between biomechanical, hormonal, and muscle parameters in the aging population, innovative treatments and preventive measures can be developed for holistically addressing the challenges faced by older women with low bone mass. A holistic approach should be employed for enhancing skeletal health and overall health outcomes in the geriatric population [31, 32].

Although our study provides valuable insights into the relationship between spinal alignment parameters and BMD in older Taiwanese women, several limitations should be acknowledged. First, this study was conducted at a single center, namely Hualien Tzu Chi Hospital in Taiwan. This limitation may affect the generalizability of our findings to broader populations, both within Taiwan and in other countries. The demographic and clinical characteristics of our study sample may not fully represent the diverse characteristics of older women with low bone mass across different regions or health-care settings. Future multicenter studies should include patients from various hospitals and regions for ensuring a more comprehensive representation of the population, thus enhancing the external validity of our findings. Second, the influence of potential instrument and measurement biases on the study results should be considered. The accuracy and precision of the imaging techniques used, particularly for measuring spinal alignment parameters, may have introduced some variability in our data. Although we employed standardized protocols for image acquisition and analysis, inherent limitations may exist in the resolution and clarity of the images, especially when using these images for assessing subtle changes in spinal curvature. Additionally, despite our efforts to maintain consistency, interobserver variability in measurements cannot be entirely ruled out, particularly for parameters requiring the manual identification of anatomical landmarks. Third, given the cross-sectional nature of our study, causal relationships between spinal alignment parameters and BMD could not be established. Longitudinal studies should be performed to determine how spinal alignment parameters change over time and the long-term effects of spinal misalignment on bone health and fracture risk. Furthermore, although we controlled for several confounding factors, other unmeasured variables may influence the relationship between spinal alignment and bone density. For instance, lifestyle factors, nutritional status, or genetic predispositions, which were not accounted for in our analysis, may play roles in both spinal alignment and bone health. Last, our study recruited only women—the findings cannot be applied to men. Given that osteoporosis and spinal alignment issues affect both genders, future studies should include male participants to provide a more comprehensive understanding of these relationships for both genders in the older adult population.

Despite the aforementioned limitations, this study still provides a comprehensive analysis of the relationships of spinal alignment parameters with BMD in older Taiwanese women with low bone mass, an under-represented population in the literature. Our findings corroborate and extend the literature on spinal sagittal balance and osteoporosis [33, 34]. The observed correlations between CL, TK, and bone mineral density as well as the strong associations between global sagittal balance parameters (SVA and GT) and BMD emphasize that these factors should be included in clinical assessments of osteoporosis risk in older women. For determining their clinical implications, the biomechanical principles underlying our findings should be considered. Spinal alignment, particularly in the sagittal plane, is vital for maintaining an efficient posture and minimizing energy expenditure. The interplay between CL, TK, LL, and global sagittal balance reflects the complexity of spinal biomechanics. With disruptions in global sagittal balance, which are often observed in older adults with decreased BMD, compensatory mechanisms are implemented, which can exacerbate postural problems. For instance, increased TK shifts the center of gravity anteriorly, increasing the muscular demand and potentially leading to fatigue, pain, and further postural deterioration. Moreover, sagittal imbalance alters the distribution of compressive and shear forces along the vertebral column, which may contribute to accelerated disc degeneration and increased vertebral fracture risk. These results suggest that spinal alignment evaluation should be incorporated into osteoporosis risk assessment and management for older women. The relationships between specific spinal parameters and BMD identified in this study can inform the development of targeted interventions, such as personalized physical therapy programs or postural training. These interventions, aimed at improving spinal alignment, can mitigate the risk of osteoporotic fractures and can improve overall function. By bridging the research gap between spinal biomechanics and bone health, the study results can guide clinical decision-making and future research in orthopedics, geriatrics, and rehabilitation medicine, underscoring the need for a holistic approach to geriatric care and spinal health.

## Conclusions

This study revealed the effects of the complex interplay between age, menopausal duration, grip strength, and sagittal spinal parameters on spinal alignment in older women with low bone mass. This study also revealed the significant correlations of clinical parameters, such as BMI and grip strength, with sagittal spinal parameters, including C7 slope and GT; these findings reflect the effects of biomechanical changes on spinal alignment. Multifaceted interventions combining hormone therapy, physical therapy, and postural management should be employed to enhance spinal alignment and overall patient wellbeing. A holistic approach considering these factors should be applied for the development of strategies for maintaining spine health and preventing related complications in older adults.

## Supporting information

S1 Data (XLSX)
